# Inhibitory Effect of *Alisma canaliculatum* Ethanolic Extract on NF-κB-Dependent CXCR3 and CXCL10 Expression in TNFα-Exposed MDA-MB-231 Breast Cancer Cells

**DOI:** 10.3390/ijms19092607

**Published:** 2018-09-03

**Authors:** Jihye Choi, Sung Shin Ahn, Yoongho Lim, Young Han Lee, Soon Young Shin

**Affiliations:** 1 Department of Biological Sciences, Sanghuh College of Life Sciences, Konkuk University, Seoul 05029, Korea; gp1686@naver.com (J.C.); wendy7130@naver.com (S.S.A.); yhlee58@konkuk.ac.kr (Y.H.L.); 2 Division of Bioscience and Biotechnology, BMIC, Konkuk University, Seoul 05029, Korea; yoongho@konkuk.ac.kr; 3 Cancer and Metabolism Institute, Konkuk University, Seoul 05029, Korea

**Keywords:** *Alisma canaliculatum*, breast cancer, migration, TNFα, IκB kinase, NF-κB, CXC motif chemokine ligand 10, CXC chemokine receptor 3

## Abstract

CXC motif chemokine ligand 10 (CXCL10) and its receptor CXC motif chemokine receptor 3 (CXCR3), play important roles in the motility of breast cancer cells. *Alisma canaliculatum* is a herb that has been used as a traditional medicine for thousands of years in Korea and China. Whether *A. canaliculatum* inhibits the motility of metastatic breast cancer cells is not clear yet. In this study, we show that *A. canaliculatum* ethanolic extract (ACE) prevented tumor necrosis factor-alpha (TNFα)-induced migration of MDA-MB-231 cells. ACE significantly attenuated TNFα-induced upregulation of CXCL10 and CXCR3 expression at the gene promoter level. Mechanistically, ACE inhibits TNFα-induced phosphorylation of inhibitor of κB (IκB) kinase (IKK), IκB and p65/RelA, leading to the suppression of nuclear translocation of p65/RelA nuclear factor kappa-B (NF-κB). Also, ACE inhibited NF-κB-dependent *CXCR3* and *CXCL10* promoter activities. These results suggest that ACE abrogates TNFα-induced migration of MDA-MB-231 breast cancer cells through down-regulation of IKK-NF-κB-dependent *CXCR3* and *CXCL1*0 expression. Our results suggest that ACE has potential as a herbal supplement for the inhibition of breast cancer metastasis.

## 1. Introduction

Breast cancer is the most common type of cancer in women worldwide. Metastasis is the uncontrolled spreading of primary tumor cells to distant organs or tissues in the body. In almost all cancers, cancer-related deaths are mainly caused by metastasis and not by the primary tumors [1]. Cellular and non-cellular components, including blood vessels, immune cells, fibroblasts, signaling molecules and extracellular matrix around primary tumor sites form specific environments, called tumor microenvironments [2]. The tumor and the surrounding microenvironment commonly interact and influence tumor progression, including invasion and metastasis [3].

Chemokines are a family of cytokines that cause cellular movements. Chemokines are classified into four main subfamilies based on the spacing of the first two cysteines adjacent to the amino terminus: CXC (or α-chemokine; X denotes any amino acids), CC (or β-chemokine), C (or γ-chemokine) and CX3C (or δ-chemokine). Cells expressing chemokine receptors on the cell surface move towards the source of the chemokine. In the tumor microenvironment, many chemokines and their receptors are intimately implicated in tumor cell migration and malignant tumor progression [4,5,6,7,8,9,10,11,12]. It is well established that CC motif chemokine ligand 5 (CCL5), also called RANTES, from mesenchymal stem cells and CCL18 from tumor-associated macrophages act on breast cancer cells to enhance cancer motility, invasion and metastasis [13,14]. Also, CXC motif chemokine ligand 10 (CXCL10) is a chemokine identified initially as interferon-gamma (IFNγ)-induced protein 10 (IP-10). CXCL10 is produced by various cell types, including monocytes, endothelial cells, keratinocytes and fibroblasts. Binding of CXCL10 to its receptor CXC motif chemokine receptor 3 (CXCR3) stimulates chemotaxis of monocytes [15], dendritic cells [16], natural killer cells [17] and type 1 helper T (Th1) lymphocytes [18,19]. Accumulating evidence indicates that CXCR3 is expressed in various cancer types, including breast cancer [20,21], ovarian carcinoma [22], glioma [23] and melanoma [24]. Also, the CXCR3 expression on breast cancer cells is involved in promoting bone metastasis [24]. Analysis of clinical data sets shows that co-expression of CXCR3 and its ligand CXCL10 is associated with early metastatic progression and increased metastatic potential in melanoma, colon carcinoma and renal cell carcinoma [25].

It has been reported that both CXCR3 and its ligand CXCL10 are highly upregulated in breast cancer cells [21,26]. In MDA-MB-435 and MCF-7 breast cancer cells, CXCR3 and CXCL10 are upregulated via the Ras signaling pathway, which contributes to breast cancer development [20], suggesting that CXCL10-CXCR3 autocrine function may play an essential role in breast cancer motility and metastasis. Therefore, strategies targeting CXCL10-CXCR3 signaling could potentially provide effective anti-metastatic therapy.

Tumor necrosis factor-alpha (TNFα) is a major inflammatory cytokine that modulates a broad range of inflammatory and immunological processes. In the tumor microenvironment, TNFα is produced by tumor cells and tumor-associated stromal cells and plays crucial roles in the expression of a variety of inflammatory cytokines and the regulation of tumor invasion and metastasis [27,28,29]. Here we found that TNFα upregulates CXCR3 and CXCL10 mRNA expression through nuclear factor kappa-B (NF-κB) activation.

*Alisma canaliculatum* is a plant native to eastern Asia, including Korea, Japan and China. It has been used as a traditional medicine for thousands of years in China as well as Korea (called Taek-sa in Korean). *A. canaliculatum* possesses various pharmacological properties, including antibacterial, antitumor and hepatoprotective activities [30,31,32,33]. However, the effects of *A. canaliculatum* on the inhibition of metastatic breast cancer cell motility have not yet been studied.

The present study aimed to evaluate whether *A. canaliculatum* ethanolic extract (ACE) inhibits the motility of metastatic breast cancer cells. Our results show that ACE prevents TNFα-induced migration of MDA-MB-231 metastatic breast cancer cells and inhibits TNFα-induced CXCR3 and CXCL10 expression through inhibition of the IκB kinase (IKK)-mediated NF-κB pathway.

## 2. Results and Discussion

### 2.1. A. canaliculatum Ethanolic Extract (ACE) Showed no Cytotoxicity against MDA-MB-231 Breast Cancer Cells

**Figure 1:**
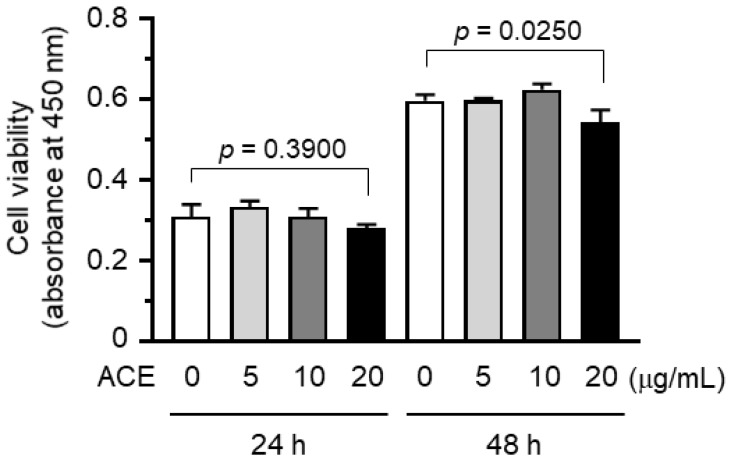
Effect of *A. canaliculatum* ethanolic extract (ACE) on cytotoxicity against MDA-MB-231 cells. The cells were treated with ACE (0, 5, 10 and 20 μg/mL) for 24 or 48 h. Cell viabilities were measured using a water-soluble tetrazolium salt which is reduced by dehydrogenase to give an orange-colored formazan dye. The absorbance of the formazan dye was measured using a microplate reader at 450 nm. Data are presented as means ± SD (*n* = 3). *p* value was analyzed by Sidak’s multiple multiple comparisons test.

To evaluate the potential anti-tumor activity of ACE, we first tested whether ACE exhibited cytotoxicity against breast cancer cells. MDA-MB-231 cells were treated with increasing concentrations of ACE. ACE did not show significant cytotoxic activity when used at a concentration of 20 μg/mL for 24 h (*p* > 0.05 with Sidak multiple comparisons test applied), although cell viability was slightly reduced when treated with the same concentration for 48 h (Figure 1). Thus, ACE showed slight cell death-inducing effects on MDA-MB-231 breast cancer cells.

### 2.2. A. canaliculatum Ethanolic Extract (ACE) Attenuates TNFα-Induced Motility of MDA-MB-231 Breast Cancer Cells

**Figure 2:**
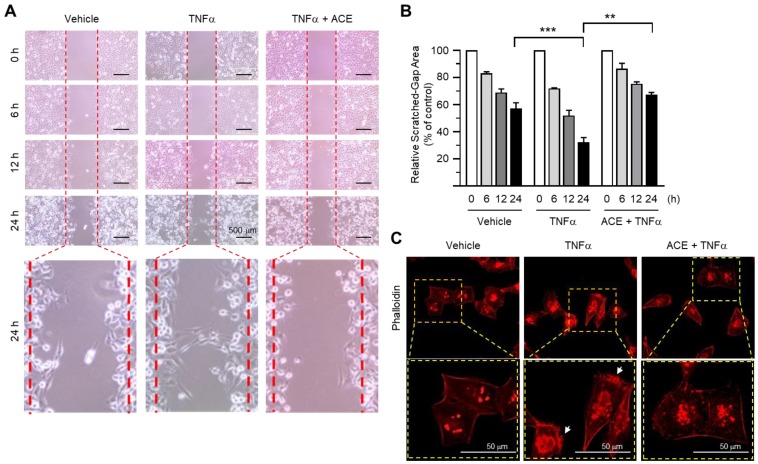
Effect of ACE on the inhibition of tumor necrosis factor-alpha (TNFα)-induced migration of MDA-MB-231 cells. (**A**) MDA-MB-231 cells were untreated (vehicle) or treated with 20 μg/mL ACE for 30 min, followed by exposure to 5 ng/mL TNFα. After 6, 12, or 24 h, representative field images were captured using an EVOS FL Auto Cell Imaging System. Dotted lines indicate the scraped boundaries at the beginning of the experiment. Scale bar, 500 μm. (**B**) Relative scratched gap area was measured using ImageJ software (National Institutes of Health, Bethesda, MD, USA). The data are presented as means ± SD (*n* = 3). *** *p* < 0.0001; ** *p* = 0.0005 by Sidak’s multiple comparisons test. (**C**) MDA-MB-231 cells were treated with vehicle (serum-free medium) or 20 μg/mL ACE for 30 min, followed by exposure to 5 ng/mL TNFα. After 12 h, the cells were incubated with rhodamine-phalloidin (1:100) for 1 h. Images were captured using an EVOS FL Auto Cell Imaging System. The arrows indicate polarized F-actin. Scale bar, 50 μm.

Cell locomotion is critical for the progression of metastasis in cancer. TNFα has been shown to increase the motility of MDA-MB-231 cells [26]. We determined whether ACE affects TNFα-induced migration of highly metastatic MDA-MB-231 cells using a scratch wound-healing assay. After creating a scratched gap in a cell monolayer, the cells were treated with either TNFα or TNFα and ACE. Consistent with a previous result [26], TNFα promoted migration of cells into the gap area when compared with the unstimulated cells (Figure 2A). However, in the presence of ACE, TNFα-induced migration of MDA-MB-231 cells was significantly reduced (*p* = 0.005 with Sidak multiple comparisons test) (Figure 2B), suggesting that ACE exhibits a property able to inhibit TNFα-stimulated migration of MDA-MB-231 breast cancer cells.

Cellular actin dynamics are associated with cell motility [34]. During cell migration, globular actin monomers (G-actin) form helical filamentous actin (F-actin). To determine the effect of ACE on actin dynamics, we examined F-actin formation using rhodamine-labeled phalloidin, a phallotoxin that binds specifically to F-actin [35]. TNFα caused morphological changes to spindle-like cells, increased polarized F-actin bundles at the cell edge and formed lamellipodia (Figure 2C). These changes disappeared in the presence of ACE. These data suggest that ACE attenuates TNFα-induced motility of MDA-MB-231 cells.

### 2.3. A. canaliculatum Ethanolic Extract (ACE) Inhibits TNFα-Induced CXCR3 mRNA Expression in MDA-MB-231 Cells

**Figure 3:**
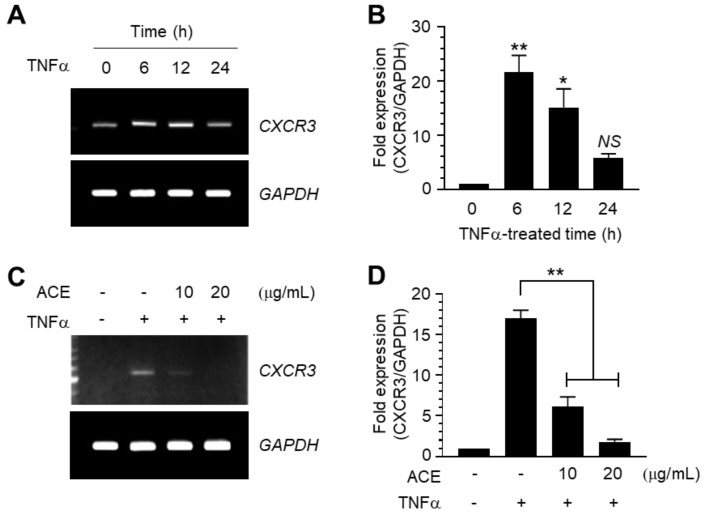
Effect of ACE on the suppression of TNFα-induced CXC motif chemokine receptor 3 (*CXCR3*) mRNA expression. (A,B) MDA-MB-231 cells were treated with 5 ng/mL TNFα for 0–24 h and total RNA was extracted. *CXCR3* mRNA levels were examined via reverse transcription polymerase chain reaction (RT-PCR, A) and quantitative real-time PCR (qPCR, B). Data are presented as means ± SD (*n* = 3). * *p* = 0.0002; ** *p* < 0.0001; compared to untreated control by Dunnett’s multiple comparisons test. Values were normalized to glyceraldehyde 3-phosphate dehydrogenase (GAPDH) mRNA levels. (C,D) MDA-MB-231 cells were treated with 5 ng/mL TNFα in the absence or presence of 20 μg/mL ACE and total RNA was extracted. CXCR3 mRNA levels were examined via RT-PCR (C) and qPCR (D). Data are presented as means ± SD (*n* = 3). ** *p* = 0.0001 by Sidak multiple comparisons test. Values were normalized to *GAPDH* mRNA levels.

CXCR3 plays an important role in regulating the chemotactic properties of activated T-lymphocytes [17]. We assessed whether ACE affects CXCR3 expression in MDA-MB-231 cells. Upon TNFα treatment, CXCR3 mRNA expression was upregulated after 6 h (Figure 3A). Quantitative real-time PCR (qPCR) analysis showed that CXCR3 mRNA expression peaked at 6 h, after which mRNA levels gradually decreased (Figure 3B). To investigate whether ACE affects CXCR3 expression, we treated MDA-MB-231 cells with TNFα for 6 h in the absence or presence of ACE. We observed that TNFα-induced *CXCR3* expression was reduced by ACE treatment, as was revealed via Reverse Transcription-Polymerase Chain Reaction (RT-PCR) analysis (Figure 3C). qPCR analysis showed that ACE significantly reduced TNFα-induced expression of *CXCR3* mRNA (*p* < 0.0001, Figure 3D).

### 2.4. A. canaliculatum Ethanolic Extract (ACE) Inhibits TNFα-Induced CXCL10 mRNA Expression in MDA-MB-231 Cells

Previously, we demonstrated that TNFα stimulates expression of the CXCR3 ligand CXCL10, also known as IP-10, in bone marrow-derived mesenchymal stem cells (BM-MSCs). TNFα-induced CXCL10 expression promotes motility and invasiveness in MDA-MB-231 breast cancer cells [26]. CXCL10 is also potentially expressed in breast cancer cells [21,36], suggesting that the CXCL10-CXCR3 autocrine pathway is relevant for breast cancer cell motility.

**Figure 4:**
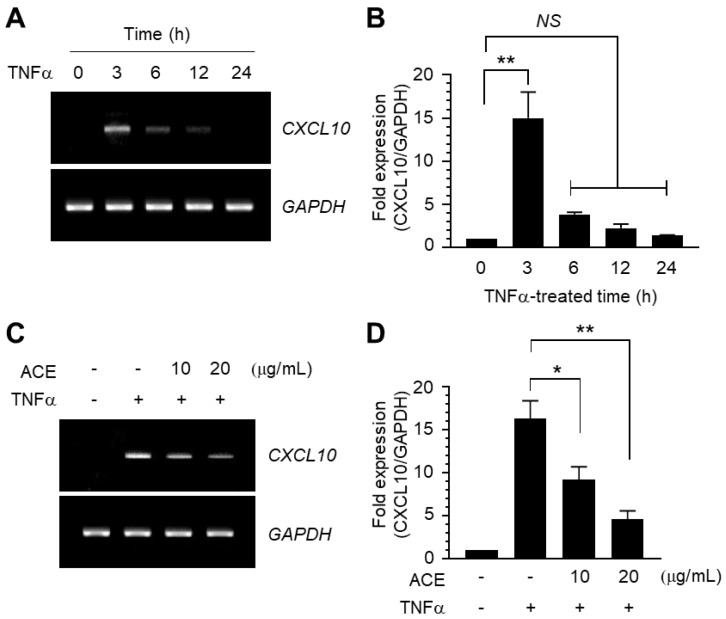
Effect of ACE on the suppression of TNFα-induced *CXCL10* mRNA expression. (**A**,**B**) MDA-MB-231 cells were treated with 5 ng/mL TNFα for 0–24 h and total RNA was extracted. CXC motif chemokine ligand 10 (*CXCL10*) mRNA levels were examined via RT-PCR (**A**) and qPCR (**B**). Data are presented as means ± SD (*n* = 3). ** *p* < 0.0001; NS: Not Significant; compared to untreated control by Dunnett’s multiple comparisons test. Values were normalized to *GAPDH* mRNA levels. (**C**,**D**) MDA-MB-231 cells were treated with 5 ng/mL TNFα in the absence or presence of 20 μg/mL ACE and total RNA was extracted. *CXCL10* mRNA levels were examined via RT-PCR (**C**) and qPCR (**D**). Data are presented as means ± SD (*n* = 3). * *p* = 0.0007; ** *p* < 0.0001 by Sidak multiple comparisons test. Values were normalized to *GAPDH* mRNA levels.

We examined whether TNFα stimulates CXCL10 expression in MDA-MB-231 cells. Following TNFα stimulation, maximal induction of *CXCL10* mRNA was detected within 3 h, as was revealed via RT-PCR (Figure 4A) and qPCR analysis (Figure 4B). We next asked whether ACE could also inhibit CXCL10 expression in MDA-MB-231 cells. Similar to CXCR3 expression, ACE inhibited TNFα-induced *CXCL10* mRNA expression, as was revealed via RT-PCR (Figure 4C). qPCR analysis showed that TNFα-induced *CXCL10* mRNA expression was reduced significantly in an ACE dose-dependent manner (all *p* < 0.001, Figure 4D). Therefore, ACE inhibits TNFα-induced expression of both CXCR3 and its ligand CXCL10 at the mRNA level in MDA-MB-231 cells.

### 2.5. Inhibitory Effects of A. canaliculatum Ethanolic Extract (ACE) on NF-κB-Mediated CXCR3 and CXCL10 Gene Promoter Activation

**Figure 5:**
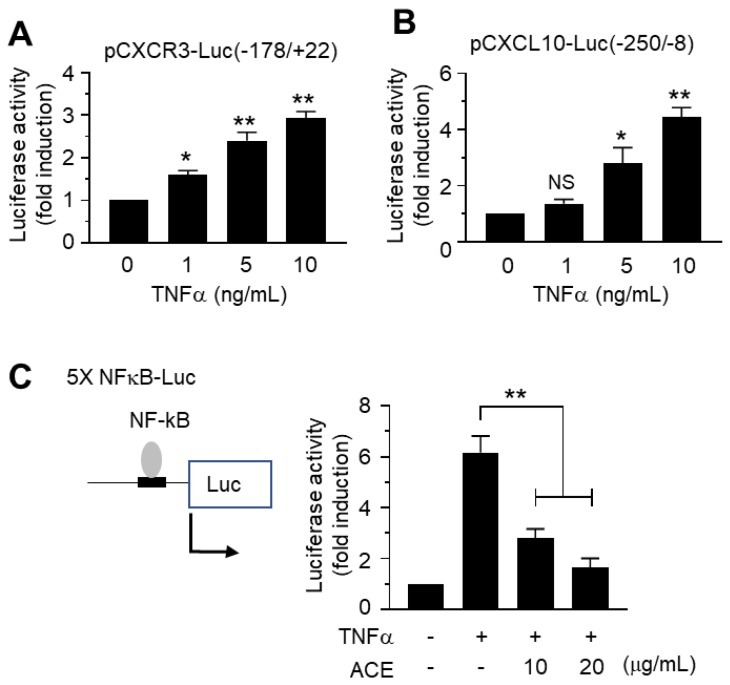
Effect of TNFα on activation of the *CXCR3* and *CXCL10* gene promoters. (**A**) MDA-MB-231 cells were transfected with 0.3 μg CXCR3 promoter reporter, pCXCR3-Luc(−178/+22). After 48 h, cells were treated with 5 ng/mL TNFα. After 8 h, cells were harvested and luciferase activities were measured. The data are presented as means ± SD (*n* = 3). * *p* = 0.0118; ** *p* < 0.001; compared to untreated control by Dunnett’s multiple comparisons test. (**B**) MDA-MB-231 cells were transfected with 0.3 μg CXCL10 promoter reporter, pCXCL10-Luc(−250/+8). After 48 h, cells were treated with 5 ng/mL TNFα and luciferase activities were measured. The data are presented as means ± SD (*n* = 3). * *p* < 0.01; ** *p* < 0.001; NS: Not Significant; compared to untreated control by Dunnett’s multiple comparisons test. (**C**) MDA-MB-231 cells were transfected with *cis*-acting 5× NF-κB-Luc plasmid. After 48 h, cells were treated with 5 ng/mL TNFα in the absence or presence of different concentrations of ACE. The data are presented as means ± SD (*n* = 3). ** *p* < 0.001 by Dunnett’s multiple comparisons test.

It is well established that TNFα stimulates NF-κB in various cell types [27,37]. NF-κB is a transcription factor that plays a central role in regulating the expression of multiple inflammatory cytokines [37,38]. We also confirmed that TNFα stimulated *CXCR3* (Figure 5A) and *CXCL10* (Figure 5B) gene promoter activities in a dose-dependent manner.

Promoter regions in the *CXCR3* and *CXCL10* genes contain p65/RelA NF-κB-binding sequences [26]. To assess whether ACE affects NF-κB-mediated transcriptional activity, we examined the effect of ACE on the inhibition of NF-κB-dependent transcription via a luciferase-driven NF-κB *cis*-acting reporter assay system. As shown in Figure 5C, TNFα caused a 6.2-fold increase in NF-κB-mediated transcription. When cells were pretreated with ACE, TNFα-induced NF-κB-dependent transcriptional activity was reduced significantly in an ACE dose-dependent manner (all *p* < 0.001).

**Figure 6:**
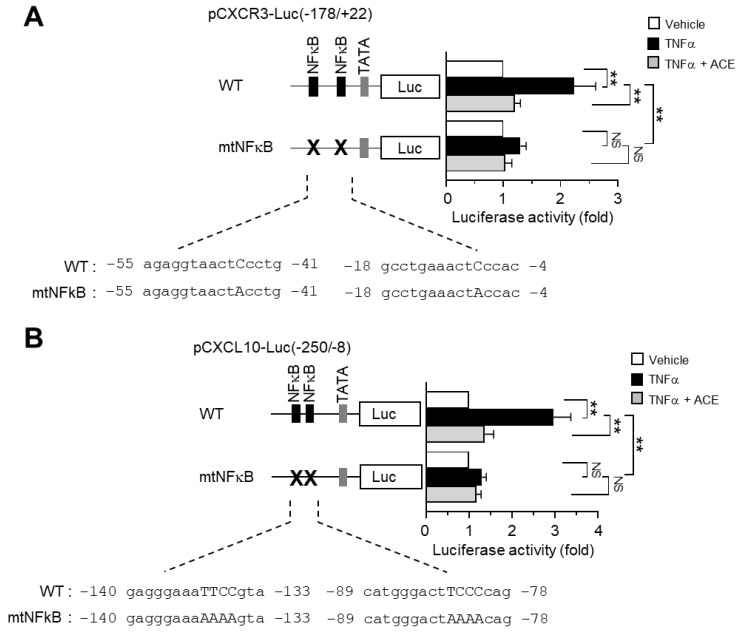
Effect of ACE on the inhibition of NF-κB-dependent *CXCR3* and *CXCL10* gene transcription. (**A**) MDA-MB-231 cells were transfected with wild-type pCXCR3-Luc(–178/+22) or the NF-κB site-mutated construct, pCXCR3-Luc(−178/+22)mtNF-κB. After 48 h, cells were treated with 5 ng/mL TNFα in the absence or presence of ACE for. After 8 h, cells were harvested and luciferase activities were measured. The data are presented are means ± SD (*n* = 3). ** *p* < 0.001; NS: Not Significant; according to Sidak multiple comparisons test. (**B**) MDA-MB-231 cells were transfected with the wild-type or NF-κB site-mutated *CXCL10* promoter construct, pCXCL10-Luc(−250/+8) or pCXCL10-Luc(−250/+8)mtNF-κB, respectively. After 48 h, cells were treated with 10 ng/mL TNFα in the absence or presence of ACE. After 8 h, cells were harvested and luciferase activities were measured. The data are presented as means ± SD (*n* = 3). ** *p* < 0.001; NS: Not Significant; according to Sidak’s multiple comparisons test.

To investigate the possibility that ACE inhibits both *CXCR3* and *CXCL10* gene expression by targeting the NF-κB pathway, we generated *CXCR3* and *CXCL10* gene promoters, both wild-type and mutants showing site-directed mutation of NF-κB -binding sites (mtNF-κB). MDA-MB-231 cells were transfected with wild-type or mtNF-κB constructs. Figure 6A shows that ACE prevented TNFα-induced *CXCR3* promoter activation in wild-type construct and that disruption of NF-κB binding sites significantly reduced TNFα inducibility (*p* < 0.001). Similarly, TNFα-induced *CXCL10* promoter activity was also inhibited by ACE treatment in wild-type construct and TNFα inducibility was significantly blocked by a mutation in the NF-κB-binding site (Figure 6B).

### 2.6. A. canaliculatum Ethanolic Extract (ACE) Suppresses NF-κB Activation via Inhibition of IKKα/β

**Figure 7:**
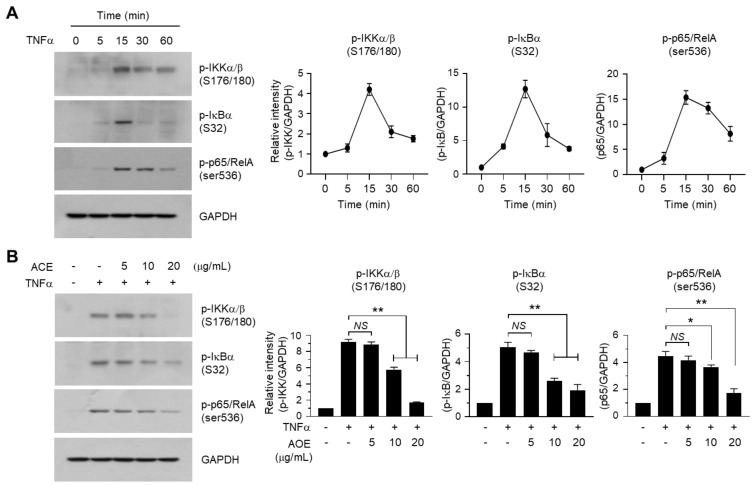
Effect of ACE on the inhibition of the TNFα-induced nuclear factor kappa-B (NF-κB) pathway. (**A**) MDA-MB-231 cells were incubated with 0.5% fetal bovine serum (FBS) for 24 h, followed by stimulation with 5 ng/mL TNFα for indicated times. Whole-cell lysates were immunoblotted with the phospho-specific antibody against inhibitor of κB (IκB) kinase (IKK)α/B (Ser176/180), IκBα (Ser32), or p65/RelA NF-κB (Ser536). The anti-GAPDH antibody was used as an internal control. The band intensities of phosphorylated (p)-IKKα/B, p-IκB and p-p65/RelA relative to GAPDH level were measured using ImageJ software. The data are presented as means ± SD (*n* = 3). (**B**) MDA-MB-231 cells were incubated with 0.5% FBS for 24 h, followed by pre-treatment with ACE (5, 10, or 20 μg/mL) 30 min before stimulation with 5 ng/mL TNFα. After 15 min, whole-cell lysates were prepared and immunoblotting was performed using the phospho-specific antibody against IKKα/B (Ser176/180), IκBα (Ser32), or p65/RelA NF-κB (Ser536). The anti-GAPDH antibody was used as an internal control. The band intensities of p-IKKα/B, p-IκB and p-p65/RelA relative to GAPDH level were measured using ImageJ software. The data are presented as means ± SD (*n* = 3). NS: Not Significant; * *p* = 0.0153; ** *p* < 0.001; according to Sidak’s multiple comparisons test.

In the resting state, NF-κB bound to IκB is inactivated in the cytoplasm. When cells are activated, IKK is phosphorylated and activated, which leads to the phosphorylation of IκB. Phosphorylated IκB is subsequently degraded, resulting in the activation of NF-κB. We confirmed that phosphorylation of IKK at Ser176/180, IκB at Ser132 and p65/RelA NF-κB at Ser536 peaked within 15 min after TNFα stimulation in MBA-MB-231 cells (Figure 7A). We next investigated whether ACE modulates the TNFα-induced NF-κB pathway. As shown in Figure 7B, treatment with ACE at concentrations greater than 10 μg/mL significantly attenuated TNFα-induced phosphorylation of IKK at Ser176/180, IκB at Ser132 and p65/RelA NF-κB at Ser536 (all *p* < 0.0001). These data suggest that ACE inhibits the TNFα-induced NF-κB signaling pathway via inhibition of IKK.

### 2.7. A. canaliculatum Ethanolic Extract (ACE) Inhibits the Translocation of NF-κB into the Nucleus of MDA-MB-231 Cells

**Figure 8:**
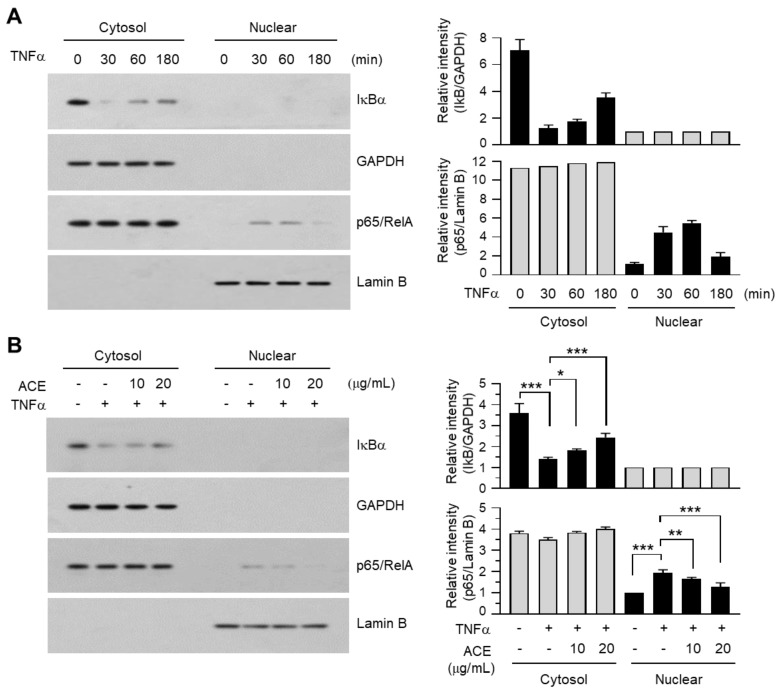
Effect of ACE on the inhibition of TNFα-induced NF-κB translocation into the nucleus. (**A**) MDA-MB-231 cells were treated with incubated with 0.5% FBS for 24 h, followed by stimulation with 5 ng/mL TNFα for indicated times. Cytosolic and nuclear fractions were immunoblotted using IκBα or p65/RelA antibodies. GAPDH was used as a cytosolic marker and Lamin B was used as a nuclear marker. The band intensities of IκBα and p65/RelA relative to GAPDH and Lamin B, respectively, were measured using ImageJ software. (**B**) MDA-MB-231 cells were treated with incubated with 0.5% FBS for 24 h, followed by pre-treatment with ACE (10 or 20 μg/mL) 30 min before stimulation with 10 ng/mL TNFα. After 30 min, cytosolic and nuclear fractions were isolated and immunoblotted using IκBα or p65/RelA antibodies. GAPDH was used as a cytosolic marker and Lamin B was used as a nuclear marker. The band intensities of IκBα and p65/RelA relative to GAPDH and Lamin B, respectively, were measured using ImageJ software. The data are presented as means ± SD (*n* = 3). * *p* = 0.0370; ** *p* = 0.0253; *** *p* < 0.001; according to Sidak multiple comparisons test.

IκB degradation in the cytoplasm and NF-κB translocation to the nucleus are recognized as markers for NF-κB-mediated transcriptional activation. To further address the effect of ACE on the inhibition of NF-κB, we measured the levels of IκB and NF-κB in the cytoplasm and nucleus. Upon TNFα stimulation, IκB levels in the cytoplasm rapidly decreased within 30 min before slowly recovering, while an increase in p65/RelA levels in the nucleus was detected within 30 min (Figure 8A). In the presence of ACE, TNFα-induced IκB degradation in the cytoplasm and accumulation of NF-κB in the nucleus were significantly prevented in a dose-dependent manner (all *p* < 0.05, Figure 8B).

**Figure 9:**
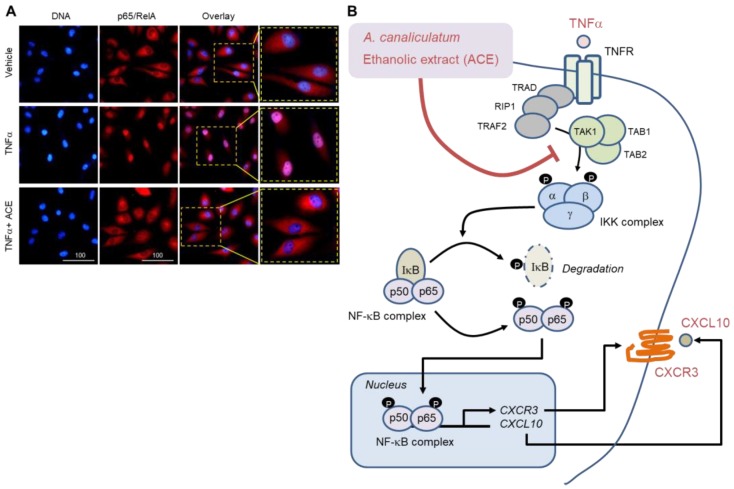
Effect of ACE on the inhibition of TNFα-induced nuclear localization of NF-κB. (**A**) MDA-MB-231 cells were treated with 10 ng/mL TNFα in the absence or presence of 20 μg/mL ACE for 30 min and then incubated with an antibody against p65/RelA NF-κB for 2 h, followed by addition of Alexa Fluor 555-conjugated (red fluorescence) secondary antibody for additional 30 min. Nuclear DNA was stained with 0.1 μg/mL Hoechst 33258 for 10 min (blue fluorescence). Fluorescence-positive cells were examined using an EVOSf1^®^ fluorescence microscope. Scale Bar, 100 μm. (**B**) Model for the inhibitory effect of ACE on TNFα-induced *CXCR3* and *CXCL10* transcription. T-bar arrow indicates inhibition and black arrows indicate activation signalings.

To further confirm the effect of ACE on NF-κB inhibition, the nuclear translocation of p65/RelA NF-κB was examined using immunofluorescence microscopy. Representative images show that p65/RelA almost entirely localized to the nucleus after TNFα stimulation, however, in the presence of ACE, this nuclear accumulation of NF-κB almost disappeared (Figure 9A). Collectively, ACE prevents TNFα-induced migration of MDA-MB-231 metastatic breast cancer cells and inhibits TNFα-induced CXCR3 and CXCL10 expression through inhibition of the IκB kinase (IKK)-mediated NF-κB pathway (Figure 9B).

Phytochemical studies have demonstrated that the major components of *A. canaliculatum* are protostane- and seco-protostane-type triterpenes such as alisols A, B and C, alisol A 24-acetate, alisol B 23-acetate, alisol C 23-acetate and alismalactone 23-acetate, as well as guaiane-type sesquiterpenes such as alismols A and B, sulfoorientalol A and orientatols A, B, C, E and F [30,39]. Of these, alisol B23 acetate exhibits antiproliferative activity via induction of apoptosis in MDA-MB-231 cells [40] and inhibition of migration and invasion through downregulation of matrix metalloproteinase (MMP)-2 and MMP-9 in human ovarian cancer cells [41]. Also, alisol F is known to inhibit inflammation via inhibition of NF-κB, mitogen-activated protein kinase (MAPK) and signal transducer and activator of transcription 3 (STAT3) in lipopolysaccharide-damaged mouse liver and RAW264.7 macrophages [42]. However, bioactive components responsible for the inhibition of IKK, which triggers NF-κB-mediated CXCR3 and CXCL10 expression, remain unknown. Further studies are needed to identify the active components that inhibit the migration of MDA-MB-231 breast cancer cells.

Metastasis is a hallmark of malignancy. Breast cancer typically metastasizes well to the bones, liver, lungs and brain [43]. The poor prognosis of breast cancer is closely related to the presence of metastasis [44]. In the early stages of tumor development, obtaining tumor cell motility and invasive potential is critical for tumor metastasis. Thus, proper control or inhibition of tumor motility is an essential strategy for the successful prevention and treatment of invasive breast cancer. Accumulating evidence demonstrates that multiple inflammatory cytokine networks around the tumor microenvironment play an amplifier in promoting metastasis of breast cancer [45]. The CXCR3 (also called as G protein-coupled receptor 9 (GPR9) and CD183) is a chemokine receptor that contributes to the progression of tumor metastasis. Its ligands, including CXCL9, CXCL10 and CXCL11, are highly expressed in breast cancer [20,21]. Also, the CXCR3 expression on breast cancer cells is involved in promoting bone metastasis [24]. Co-expression of CXCR3 and its ligand CXCL10 is associated with the increased metastatic potential of melanoma, colon carcinoma and renal cell carcinoma [25], suggesting that CXCL10-CXCR3 autocrine signaling functions as a promoter of cancer motility and metastasis. Thus, targeting CXCL10-CXCR3 autocrine signaling can provide an effective strategy for the treatment of invasive breast cancer.

*A. canaliculatum* has been widely used in traditional medicine for thousands of years in China as well as Korea because of its various pharmacological properties [30,31,32,33]. Also, here we have shown that *A. canaliculatum* ethanolic extract (ACE) reduces the expression of CXCL10 and its receptor CXCR3 through the inhibition of the IκB kinase (IKK)-mediated NF-κB pathway, which play an important role in the migration of MDA-MB-231 metastatic breast cancer cells. Based on these results, we suggest that *A. canaliculatum* has potential as a herbal supplement for breast cancer prevention and treatment. Further studies are needed to determine the inhibitory effect of breast cancer metastasis in animal models to validate efficacy in vivo. Because ACE has weak cytotoxicity, it can be used in combination with other anti-cancer drugs for effective treatment of invasive breast cancer.

## 3. Materials and Methods

### 3.1. Preparation of A. canaliculatum Ethanolic Extract (ACE)

*A. canaliculatum* root was purchased from the Kyungdong traditional medicine market in Seoul, Korea. The ethanolic extract was prepared using 1 kg of dried root and 3 L ethanol at room temperature in a dark room. After filtration, the extract was concentrated under reduced pressure in a rotary evaporator (Eyela, Tokyo, Japan). The residue was re-suspended in distilled water (1 L) and freeze-dried at −90 °C and 0.1 atm for 60 h. This freeze-dried ethanolic extract was used in the biological experiments.

### 3.2. Cells and Reagents

MDA-MB-231 human breast cancer cells were obtained from American Type Culture Collection (ATCC, Rockville, MD, USA) and cultured in Dulbecco’s modified Eagle’s medium (DMEM) supplemented with 10% fetal bovine serum (CellGro/Corning, Manassas, VA, USA). Antibodies against phospho (p)-IKKα/β (Ser176/180), p-IκBα (Ser32), IκB and p-p65/RelA (Ser536) were obtained from Cell Signaling Technology (Beverly, MA, USA), while glyceraldehyde phosphate dehydrogenase (GAPDH), p65 NF-κB and Lamin B were obtained from Santa Cruz Biotechnology (Santa Cruz, CA, USA). Alexa Fluor 488-conjugated secondary antibodies were purchased from Invitrogen (Carlsbad, CA, USA). The firefly and *Renilla* Dual-Glo^™^ Luciferase Assay System was obtained from Promega (Madison, WI, USA). Hoechst 33258 was purchased from Sigma-Aldrich (St. Louis, MO, USA).

### 3.3. Cytotoxicity Assay

Cell viability was measured with a Cell Counting Kit-8 (CCK-8; Dojindo Molecular Technologies, Gaithersburg, MD, USA), according to the manufacturer’s instructions. Briefly, exponentially growing cells (3 × 10^3^ cells/sample) were exposed to either the vehicle or different concentrations of ACE (0, 5, 10 and 20 μg/mL) for 24 or 48 h, followed by the addition of CCK-8 solution, containing water-soluble tetrazolium salt WST-8 (2-(2-methoxy-4-nitrophenyl)-3-(4-nitrophenyl)-5-(2,4-disulfophenyl)-2H-tetrazolium, monosodium salt), for an additional 1 h. Colorless WST-8 is reduced by dehydrogenase in cells to produce an orange-colored product, WST-8 formazan dye. The amount of WST-8 formazan dye is proportional to the number of living cells. The absorbance of WST-8 formazan was measured at 450 nm using an Emax Endpoint ELISA Microplate Reader (Molecular Devices, Sunnyvale, CA, USA).

### 3.4. Scratch Wound-Healing Assay

The motility of MDA-MB-231 cells was measured using a cell scratch wound-healing assay as described previously [46]. Briefly, MDA-MB-231 cells were grown in 6-well plates. After reaching confluence, the monolayer was scratched using a pipette tip dragged across the center of the well to create a wound-like gap, followed by washing with phosphate-buffered saline (PBS) to remove cell debris. Cells were treated with serum-free medium (vehicle) or 10 ng/mL TNFα in the absence or presence of 20 μg/mL ACE. After 6, 12 and 24 h, cells were photographed using a Nikon E500 camera (Nikon Corporation, Tokyo, Japan). Relative scratched gap area was measured using ImageJ version 1.52a software (National Institute of Health, Bethesda, MD, USA). The gap area of the control group (0 h) was designated as 100%.

### 3.5. Actin Reorganization

MDA-MB-231 cells grown on glass coverslips were treated with 10 ng/mL TNFα in the absence or presence of 20 μg/mL ACE for 12 h, fixed in 4% paraformaldehyde and then permeabilized with 0.3% Triton X-100. Actin rearrangement was determined using the rhodamine phalloidin-based F-Actin Visualization Biochem Kit^TM^ (Cytoskeleton Inc., Denver, CO, USA), according to the manufacturer’s instructions. Polymerized F-actin was analyzed using an EVOSf1^®^ fluorescence microscope (Advance Microscopy Group, Bothell, WA, USA).

### 3.6. Quantitative Real-Time PCR (qPCR) Analysis

Total RNA was extracted using a TRIzol RNA extraction kit (Invitrogen, Carlsbad, CA, USA). The first-strand cDNA was synthesized using an iScript cDNA synthesis kit (Bio-Rad, Hercules, CA, USA). Quantitative real time-PCR (qPCR) was performed using an iCycler iQ^™^ system (Bio-Rad) according to the manufacturer’s recommendations. The reaction mixture (20 μL) contained 10 μL TaqMan-iQ supermix Kit (Bio-Rad). Gene-specific primer sequences are as Table 1. 

### 3.7. Construction and Mutagenesis of the CXCR3 and CXCL10 Promoter Reporters

The methods used for the construction of the *CXCL10* gene promoter-driving luciferase reporter, wild-type pCXCL10-Luc(−250/+8)) and disruption of the NF-κB-binding site by site-directed mutagenesis, pCXCR3-Luc(−178/+22)mtNF-κB, are described elsewhere [26].

For the generation of *CXCR3* gene promoter-driving luciferase reporter constructs, the *CXCR3* promoter fragment spanning nucleotides −178 to +22 upstream of the transcription start site was synthesized from human genomic DNA (Promega, Madison, WI, USA) via PCR using the primers 5′-ggtaccCATCCTCTGCCAGCTTTTCT-3′ (forward primer) and 5′-agatctCTTTGGTGCTTGTGGTTGGA-3′ (reverse primer). Small letters indicate inserted KpnI and BglII restriction sites. The PCR products ligated into a T&A vector (RBC Bioscience, Taipei county, Taiwan) were digested by KpnI and BglII and cloned into the KpnI and BglII sites of the pGL4-basic vector (Promega), yielding pCXCR3-Luc(−178/+22). Site-specific mutation of two NF-κB binding sites, mtNF-κB(I) and mtNF-κB(II), was performed using an EZchange Site-directed Mutagenesis Kit (Enzynomics, Daejeon, Republic of Korea), using the −178/+22 construct as a template plasmid. Primer sequences used to generate point mutations were as Table 2.

RT-PCR was carried out by annealing at 55 °C for 25 cycles. The point mutation was verified by DNA sequencing (Macrogen, Seoul, Korea).

### 3.8. Gene Promoter Reporter Activity Assay

MDA-MB-231 cells were seeded onto 12-well plates and transfected with 0.3 μg of the *CXCR3* or *CXCL10* promoter construct using Lipofectamine^™^ 2000 (ThermoFisher Scientific, Waltham, MA, USA) according to the manufacturer’s instructions. At 48 h post transfection, cells were treated with TNFα in the absence or presence of ACE. Luciferase activity was determined with a luminometer (Centro LB960; Berthold Technologies, Bad Wildbad, Germany). The relative amount of luciferase activity in the untreated cells was designated as 1.

### 3.9. Immunoblot Analysis

Cells were extracted with 20 mM HEPES (pH 7.2) containing 1% Triton X-100, 10% glycerol, 150 mM NaCl, 10 μg/mL leupeptin and 1 mM phenylmethylsulfonyl fluoride (PMSF). The protein extracts (20 μg each) were separated via 10% sodium dodecyl sulfate-polyacrylamide gel electrophoresis (SDS-PAGE) and transferred to nitrocellulose membranes [47]. Primary and secondary antibodies were added and developed using an enhanced chemiluminescence detection system (GE Healthcare, Piscataway, NJ, USA). Relative band intensity was quantified using ImageJ version 1.52a software (National Institute of Health). Relative band intensity was expressed as a ratio to GAPDH (cytosolic marker) or Lamin B (nuclear marker).

### 3.10. NF-κB-Dependent Transcriptional Activity

After 24 h post transfection with 0.1 μg of the 5 × NF-κB-Luc plasmid, MDA-MB-231 cells were treated with TNFα (10 ng/mL) in the absence or presence of ACE (5, 10, or 20 μg/mL). After 8 h, luciferase activities were measured using a Centro LB960 luminometer (Berthold Technologies, Bad Wildbad, Germany).

### 3.11. Nuclear Extraction

MDA-MB-231 cells were treated with TNFα in the absence or presence of ACE. GAPDH or Lamin B was used as a cytosolic or nuclear marker, respectively. After harvesting, cells were rinsed with cold PBS, scraped, collected by centrifugation, re-suspended in lysis buffer (50 mM Tris pH 8.0, 0.5% Triton X-100, 10 μg/mL leupeptin and 1 mM PMSF), transferred to a 1.5 mL Eppendorf tube and kept on ice for 20 min. Nuclei were collected by centrifugation (13,200 rpm, 30 min at 4 °C) and washed in lysis buffer (50 mM Tris pH 8.0, 0.5% Triton X-100, 10 μg/mL leupeptin and 1 mM PMSF). Nuclei were re-suspended in extraction buffer (50 mM Tris pH 8.0, 0.5% Triton X-100, 0.4 M NaCl, 10 μg/mL leupeptin and 1 mM PMSF) for 30 min and the suspension was centrifuged (13,200 rpm, 30 min at 4 °C).

### 3.12. Immunofluorescence Microscopic Analysis

MDA-MB-231 cells cultured on coverslips were treated with 5 ng/mL TNFα for 15 min. The cells were fixed in 4% (*w*/*v*) paraformaldehyde and permeabilized using 0.1% (*v*/*v*) Triton X-100, as described previously [48]. To detect the localization of NF-κB, we incubated with anti-p65/RelA antibody for 2 h, followed by addition of Alexa Fluor 555-conjugated (red staining) secondary antibody for 30 min. Nuclear DNA (blue staining) was stained with 1 μg/mL Hoechst 33258 (Sigma-Aldrich). Stained cells were examined using an EVOSf1^®^ fluorescence microscope (Advance Microscopy Group, Bothell, WA, USA).

### 3.13. Statistical Analysis

All data are presented as means ± standard deviation (SD) of at least three independent experiments. Statistical analysis was carried out using one-way analysis of variance (ANOVA) followed by Sidak multiple comparisons test using GraphPad Prism version 7.04 software (GraphPad Software Inc., La Jolla, CA, USA). A value of *p* < 0.05 was considered statistically significant.

## 4. Conclusions

Multiple chemokines and their receptors are closely implicated in tumor cell migration and malignant tumor progression. CXCR3-CXCL10 autocrine function plays an important role in breast cancer motility and metastasis. Therefore, targeting CXCL10-CXCR3 signaling could potentially provide an effective strategy for anti-metastatic therapy. *A. canaliculatum* ethanolic extract (ACE) inhibits TNFα-induced migration of MDA-MB-231 metastatic breast cancer cells and prevents TNFα-induced CXCR3 and CXCL10 expression through inhibition of the IκB kinase (IKK)-mediated NF-κB pathway(Figure 9B). ACE could be used as a potential supplement to inhibit invasion and metastasis of breast cancer cells.

## Figures and Tables

**Table 1:** Gene specific primer sequences.

Gene Name	Primer
*CXCL10*	Forward, 5′-AGCAAGGAAAGGTCTAAAAGATCTCC-3′
	Reverse, 5′-GGCTTGACATATACTCCATGTAGGG-3′
	TaqMan probe, 5′-FAM-AGGCAGCCTCTGTGTGGTCCATCCTT-BHQ-3′
*CXCR3*	Forward, 5′-GCTCTGAGGACTGCACCATTG-3′
	Reverse, 5′-TGAAGTTTTAGTTTCCAAATGAGAAGGG-3′
	TaqMan probe, 5′-FAM-CTGCCAAGCCCCATCCTGCCGCC-BHQ-3′
*GAPDH*	Forward, 5′-TCGACAGTCAGCCGCATCTTC-3′
	Reverse, 5′-CGCCCAATACGACCACCTCCG-3′
	TaqMan probe, 5′-Yakima Yellow TM-CGTCGCCAGCCCAGCCACGC-BHQ-1-3′

The specificity of qPCR was verified by melting curve analysis. GAPDH was used to normalize the RNA in tested samples. Reverse transcription-PCR (RT-PCR) was performed by annealing at 55 °C for 25 cycles.

**Table 2:** The primer sequences used for site-specific mutations.

Mutant Name	Primer
*mtNF-κB(I)*	Forward, 5′-CCTGGAAGAGGCTGCTGC-3′
	Reverse, 5′-TAGTTACCTCTACCAGACCTCCCTAAA-3′
*mtNF-κB(II)*	Forward, 5′-CCACTTCCTCTGTGACTGCAG-3′
	Reverse, 5′-TAGTTTCAGGCAGTTCTCAGCAG-3′

## Abbreviations

ACE	*A. canaliculatum* ethanolic extract
TNFα	Tumor necrosis factor-alpha
IκB	Inhibitor of κB
IKK	IκB kinase
NF-κB	Nuclear factor kappa-B paired box gene 3
CXCL10	CXC motif chemokine ligand 10
CXCR3	CXC motif chemokine receptor 3
RT-PCR	Reverse transcription-polymerase chain reaction
qPCR	quantitative real time-PCR
GAPDH	Glyceraldehyde phosphate dehydrogenase
